# Randomized, Triple-Blinded, Placebo-Controlled Trial of SA3X (Spilanthes acmella) for the Management of Erectile Dysfunction

**DOI:** 10.7759/cureus.23989

**Published:** 2022-04-09

**Authors:** Nabnita Patnaik, Kumar Guru Mishra, Nihar Ranjan Pradhan

**Affiliations:** 1 Obstetrics and Gynecology, All India Institute of Medical Sciences, Bibinagar, Hyderabad, IND; 2 Community and Family Medicine, All India Institute of Medical Sciences, Bibinagar, Hyderabad, IND; 3 Vascular and Endovascular Surgery, Apollo Hospitals, Hyderabad, IND

**Keywords:** men’s health, intention-to-treat analysis, sexual behaviour, sexual health, spilanthol, erectile dysfunction, spilanthes acmella

## Abstract

Introduction

*Spilanthes acmella* has been used as an aphrodisiac in India and other countries. However, studies concerning humans have been limited. This randomized controlled trial was carried out to evaluate the effect of SA3X capsules containing 500 mg of *S. acmella* on sexual function domain scores in sexually active men with symptoms of erectile dysfunction (ED) using the Men’s Sexual Health Questionnaire (MSHQ).

Materials and methods

This triple-blind, placebo-controlled, parallel-group was conducted at two centres in Hyderabad and Secunderabad from May to December 2021. Patients were randomized 1:1 to SA3X therapy or placebo for one month along with an observational cohort. The change of MSHQ score and its subdomains from baseline to month 1 (primary endpoint) and one-month post-treatment (secondary outcome) was assessed using a mixed model repeated measures analysis. Additional secondary outcomes measured were the change in the International Index of Erectile Function (IIEF) and duration of penile erection. Safety was evaluated.

Results

The intention-to-treat population included 448 patients (152 - SA3X therapy; 146 - placebo; 150 - observational cohort). A significant increase was observed with SA3X therapy versus placebo on the total MSHQ score (17.24 vs 4.72; SE: 2.11, 1.98; P<0.001) along with the sub-domains at the end of one month of therapy. At one-month post-treatment, the increase in MSHQ score with SA3X therapy was significant (18.48 vs 3.78; SE 2.81, 1.39; P<0.001). The IIEF scores and duration of penile erection also increased significantly in the SA3X therapy group. Dysgeusia (3.94%) was the most common drug-related adverse effect. No serious adverse effects were noted.

Conclusion

SA3X was concluded to be safe and effective as a potential treatment for ED.

## Introduction

A plentitude of herbal medicines claims to improve sexual health, restore erection and boost the vitality of males and females alike [1]. Despite recent advancements in pharmaceutical therapies, the satisfaction of consumers seems diminished owing to higher costs and adverse events (AEs), steering them to opt for herbal/complementary formulations [1,2]. *Spilanthes acmella* (Akarkara) is one such product among alternative/complementary medicines currently in demand and being used by a specific set of people for its alleged benefits as an aphrodisiac [3].

*S. acmella* has diverse biological and pharmacological effects due to spilanthol (N-isobutyl-2E, 6Z, 8E-decatrienamide), an active constituent of the herb [4-6]. It has been used as a folk medicine for toothache in many parts of India and worldwide. Also, it exhibits antioxidant, anti-microbial, analgesic, neuroprotective, anti-inflammatory, insecticidal, anti-larvicidal, and anti-teratogenic properties [6-10]. Besides these, few murine studies have substantiated spilanthol to be a potent aphrodisiac resulting in improved male sexual performance, which has been reflected via an increased duration of penile erection along with augmented frequencies of mounting, intromission, and ejaculation [3,8].

A few industries, like Stiriti Ayur Therapies Pvt. Ltd., have recently introduced SA3X capsules containing 500 mg of *S. acmella* extract standardized to 3.5% spilanthol, yielding 17.5 mg of spilanthol. Regardless of the large consumption of such formulations, these products have not been subjected to rigorous human trials, which paved the way for this randomized controlled trial (RCT) with the objectives of evaluating the effect of SA3X capsules consumption for one month and its impact thereafter on the sexual function domain scores in sexually active men using Male Sexual Health Questionnaire (MSHQ).

## Materials and methods

This was a hospital-based, triple-blind, placebo-controlled, parallel-group study conducted at two centres of Apollo Hospitals in Hyderabad and Secunderabad. The RCT complies with the Consolidated Standards of Reporting Trials (CONSORT) statement for reporting RCTs [11].

The ethical approval for the study has been taken Institutional Ethics Committee - Biomedical Research, Apollo Hospitals, Hyderabad (AHJ-ACD-045/03-21), and the study was registered at Clinical Trials Registry India (CTRI) in May 2021 (CTRI/2021/05/033694). The enrollment started in May 2021 and recruitment was completed in December 2021. The research was conducted in accordance with the Helsinki Declaration. A total of 448 participants fulfilling the eligibility criteria below were included in the study. Table 1 highlights the inclusion and exclusion criteria of the participants. 

**Table 1:** Inclusion and exclusion criteria PDE: Phosphodiesterase; ED: Erectile Dysfunction

Inclusion Criteria	Exclusion Criteria
Age:18–45 years	Current use of PDE inhibitors (e.g. sildenafil etc.)/ testosterone supplements/ immunosuppressants (including, but not limited to, antibiotics, non-steroidal anti-inflammatory drugs, oral/injectable corticosteroids).
Self-reported symptoms of ED presenting to the out-patient departments.	Patients with an active urinary tract infection or prostatitis
Willing to participate in 2 months of supervised drug trial.	Current or lifetime diagnosis of bipolar disorder, personality disorder or eating disorder.
	History of alcohol or substance abuse disorder (abuse/ dependence) within 6 months.
	Presence or history of following illness: serious allergic reaction, diabetes mellitus, uncontrolled hypertension, unstable cardio-vascular, pulmonary, renal, hepatic, endocrine, hematological or active infectious diseases, stroke, cancer, auto-immune disease.
	Any major episode of infection requiring hospitalization or treatment with intravenous antibiotics within 4 weeks.

Recruitment procedure

**Figure 1:**
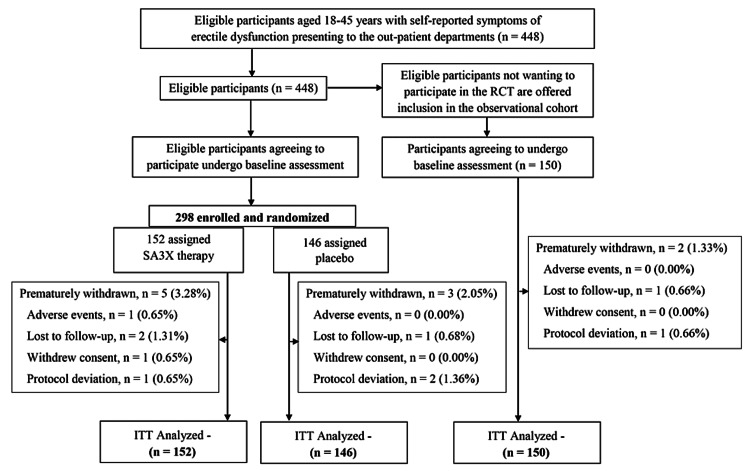
Flowchart showing study conduction process RCT: Randomized Controlled Trial; ITT: Intention to Treat

The overall trial flow is outlined in Figure 1. Patients attending the out-patient departments of Apollo Hospitals complying with the eligibility criteria were invited to participate in the RCT. Oral and written information about the study was provided by the examining doctor during consultation at the hospital. For subjects unable to decide on participation, it was recommended to take at least 24 hours to consider participation in the study. Patients who fulfilled all eligibility criteria, but did not wish to participate in the randomized study were enrolled in an observational cohort with the same self-reported questionnaires. Written informed consent was obtained from each of the participants.

Randomization procedure and concealment of allocation

Patients fulfilling eligibility criteria and willing to participate were randomized into two groups after baseline assessment (1:1 allocation ratio): the intervention group where they received SA3X capsules or the control group receiving a placebo. A priori, an independent administrator had prepared a computer-generated randomization schedule in random-sized permuted blocks of variable size, randomly varying from two, four, or six participants. Stratification factors in the randomization were age and history of addiction. The treatment allocation scheme was prepared by the administrator and concealed in opaque sealed envelopes. The envelopes were only accessible by another study coordinator who opened them after informed consent and baseline measures had been obtained and were responsible for dispensing the drugs to the participants in sealed containers.

Blinding

Investigators were not involved in the dispensing of the study drugs. Patients, medical staff, and investigators were masked for group assignment. The researchers conducting the outcome assessments and data analysis were also blinded. Blinded results from the analyses were presented to all authors, who agreed on two possible written interpretations before the data manager unblinded the randomization code.

Procedure

Patients in the intervention arm received SA3X capsules supplied by Stirti Ayur Therapies Pvt. Ltd. The patients consumed the capsules once daily at night post-dinner for a total of 30 days. Patients were prescribed other medicines for other clinical conditions they might have had in the usual way. Patients in the placebo group received placebo capsules in a similar dosing scheme. Data were collected at baseline. All participants attended a face-to-face assessment comprising anthropometric measures and questionnaire administration. A follow-up assessment took place at end of 30 days of drug administration and was similar to baseline data collection. At one-month post-treatment completion, participants were assessed again.

Outcome

The primary outcome was the between-group mean difference in total MSHQ score between the group randomized to receive SA3X capsules and the group randomized as control from baseline to day-30 of treatment. The MSHQ is a 25-item self-administered questionnaire. It encompasses three scales: Erection scale (three items), Ejaculation scale (seven items), Satisfaction scale (six items), and nine additional items addressing sexual activity, time since last sexual encounter, level, and changes in sexual activity, and bother associated with sexual dysfunction. For the three scales, the total score of 16 questions ranges from seven to 80 and higher scores indicate better sexual function [12]. The change from baseline in total MSHQ score a one-month post-treatment was assessed as a secondary outcome. Additional secondary outcomes included the mean change in the International Index of Erectile Function (IIEF) total score - a 15-question validated, multi-dimensional, self-administered questionnaire that has been found useful in the clinical assessment of ED and treatment outcomes in clinical trials. A score of 0-5 is awarded to each of the 15 questions that examine the five main domains of male sexual function: erectile function (six items), orgasmic function (two items), sexual desire (two items), intercourse satisfaction (three items), and overall satisfaction (two items) [13-15]. The participants were also asked to report the duration (in minutes) for which they are able to retain the penile erection without being flaccid in the first instance of sexual arousal for the last seven days and the average duration was calculated at baseline and all follow-ups.

Data management and monitoring

A unique participant ID number was provided to each recruited participant. Each stage of data collection for a given participant was tracked to determine their (anonymized) present status in the study, and assessment and other follow-up dates may be predicted. To ensure security before, during, and after the trial, this information was stored on a secure, password-protected database. Web-based data entry technologies were used to upload anonymized data from assessments to the safe, password-protected database, with paper copies being transcribed as needed. Monthly meetings of the Data and Safety Monitoring Committee were held. Every medication error was immediately reported to the committee.

Adverse events

At all follow-ups, AEs and serious AEs (SAE) were recorded by asking patients about potential AEs using open-probe questioning to ensure that all AEs were documented. Any unfavourable experience during treatment or follow-up that resulted in contact with the healthcare system was referred to as an AE. An SAE was defined as any untoward medical event that (1) resulted in death, (2) was life-threatening, (3) necessitated inpatient hospitalization, (4) resulted in persistent or significant disability, or (5) necessitated intervention to prevent permanent impairment or damage that occurred during the study period [16]. All AEs and SAEs were followed until resolution.

Statistical analysis

The sample size was based on the anticipated change in the study’s primary outcome measure of the total MSHQ score. The study was powered to detect a six‐unit treatment difference. To detect this difference, 140 patients in each of the arms - intervention vs control - were needed (assuming a common SD of 18, power=80%, alpha level=0.05, and lost to follow-up of 20%). Patients for the observational cohort were included consecutively until inclusion in the RCT has been completed or until 150 patients have been included. A mixed model repeated measures (MMRM) analysis was used to analyse the change in MSHQ score from baseline. The change from baseline in total MSHQ score with SA3X therapy vs placebo at 30 days was the primary treatment comparison for the intention-to-treat (ITT) population. 95% CI was calculated for the treatment difference in the change from baseline to 30 days post-therapy and the significance level was fixed at 0.05. The MMRM analysis method was also used to compare the secondary outcome, i.e., change from baseline in the total MSHQ at one-month post-treatment along with all domains of MSHQ, and scores on the IIEF. The proportion of patients with AEs and SAEs was compared between treatment groups using Chi-square’s test and Fisher’s exact test wherever applicable.

## Results

Study population and patient demographics

The ITT population included 448 patients (152 in the SA3X therapy group, 146 in the placebo group, and 150 in the observational cohort). Demographic and baseline characteristics were similar across the three groups and indicative of a population with alleviated sexual health (Table 2).

**Table 2:** Summary of demographics and baseline characteristics (ITT population) ITT: Intention to treat; MSHQ: Male Sexual Health Questionnaire; IIEF: International Index of Erectile Function

		SA3X therapy (N = 152)	Placebo (N = 146)	Observational Cohort (N = 150)	Total (N = 448)
Age (years)	Mean ± SD	33.4 ± 6.49	32.1 ± 5.98	32.8 ± 6.01	32.7 ± 6.16
	Median (Range)	33 (25 – 45)	32 (23 – 45)	33 (24 – 45)	33 (23 – 45)
Height (cm)	Mean ± SD	174.57 ± 10.17	174.12 ± 10.32	173.21 ± 9.76	173.97 ± 10.07
	Median (Range)	174.20 (164.50 – 190.00)	173.50 (165.00 – 189.00)	173.90 (164.00 – 189.00)	174.10 (164.00 – 190.00)
Weight (kg)	Mean ± SD	59.78 ± 5.65	60.54 ± 4.89	60.21 ± 5.21	60.17 ± 5.24
	Median (Range)	60.20 (50.00 – 75.00)	61.00 (52.00 – 74.00)	60.80 (50.50 – 75.00)	60.50 (50.00 – 75.00)
Baseline total MSHQ score	Mean ± SD	20.11 ± 5.52	20.27 ± 5.19	20.33 ± 4.95	20.25 ± 5.36
	Median (Range)	21 (10 - 34)	21 (10 - 34)	20 (10 - 34)	21 (10 - 34)
Baseline total erection score	Mean ± SD	3.61 ± 1.82	3.42 ± 1.97	3.54 ± 1.76	3.52 ± 1.81
	Median (Range)	5 (3 – 8)	5 (3 - 8)	5 (3 - 8)	5 (3 - 8)
Baseline total ejaculation score	Mean ± SD	7.43 ± 2.98	7.98 ± 2.45	8.12 ± 2.74	7.89 ± 2.71
	Median (Range)	7 (1 – 15)	8 (1 - 15)	7 (1 - 15)	7 (1 - 15)
Baseline total satisfaction score	Mean ± SD	9.07 ± 3.02	8.87 ± 2.76	8.67 ± 2.45	8.84 ± 2.74
	Median (Range)	9 (6 - 11)	8 (6 - 11)	8 (6 - 11)	8 (6 - 11)
Baseline total bother score	Mean ± SD	2.13 ± 0.87	2.43 ± 0.23	2.57 ± 0.71	2.37 ± 0.58
	Median (Range)	2 (1 – 3)	2 (1 – 3)	2 (1- 3)	2 (1 – 3)
Baseline total activity score	Mean ± SD	3.76 ± 1.65	4.01 ± 1.02	3.89 ± 1.21	3.85 ± 1.23
	Median (Range)	4 (3 – 6)	4 (3 – 7)	4 (3 – 6)	4 (3 – 7)
Baseline total desire score	Mean ± SD	6.98 ± 2.16	7.01 ± 1.96	6.85 ± 2.23	6.89 ± 2/04
	Median (Range)	7 (4 – 10)	7 (4 – 10)	7 (4 – 10)	7 (4 – 10)
Baseline IIEF score	Mean ± SD	17.56 ± 4.84	17.26 ± 5.02	18.02 ± 4.27	17.61 ± 4.65
	Median (Range)	18 (10 – 30)	17 (9 – 30)	18 (10 – 30)	18 (9 – 30)
Duration of penile erection (minutes)	Mean ± SD	1.67 ± 1.01	1.98 ± 0.97	1.88 ± 0.87	1.85 ± 0.92
	Median (Range)	2 (1 – 4)	2 (1 – 4)	2 (1 – 4)	2 (1 – 4)

Efficacy results

Primary Efficacy Results

**Figure 2:**
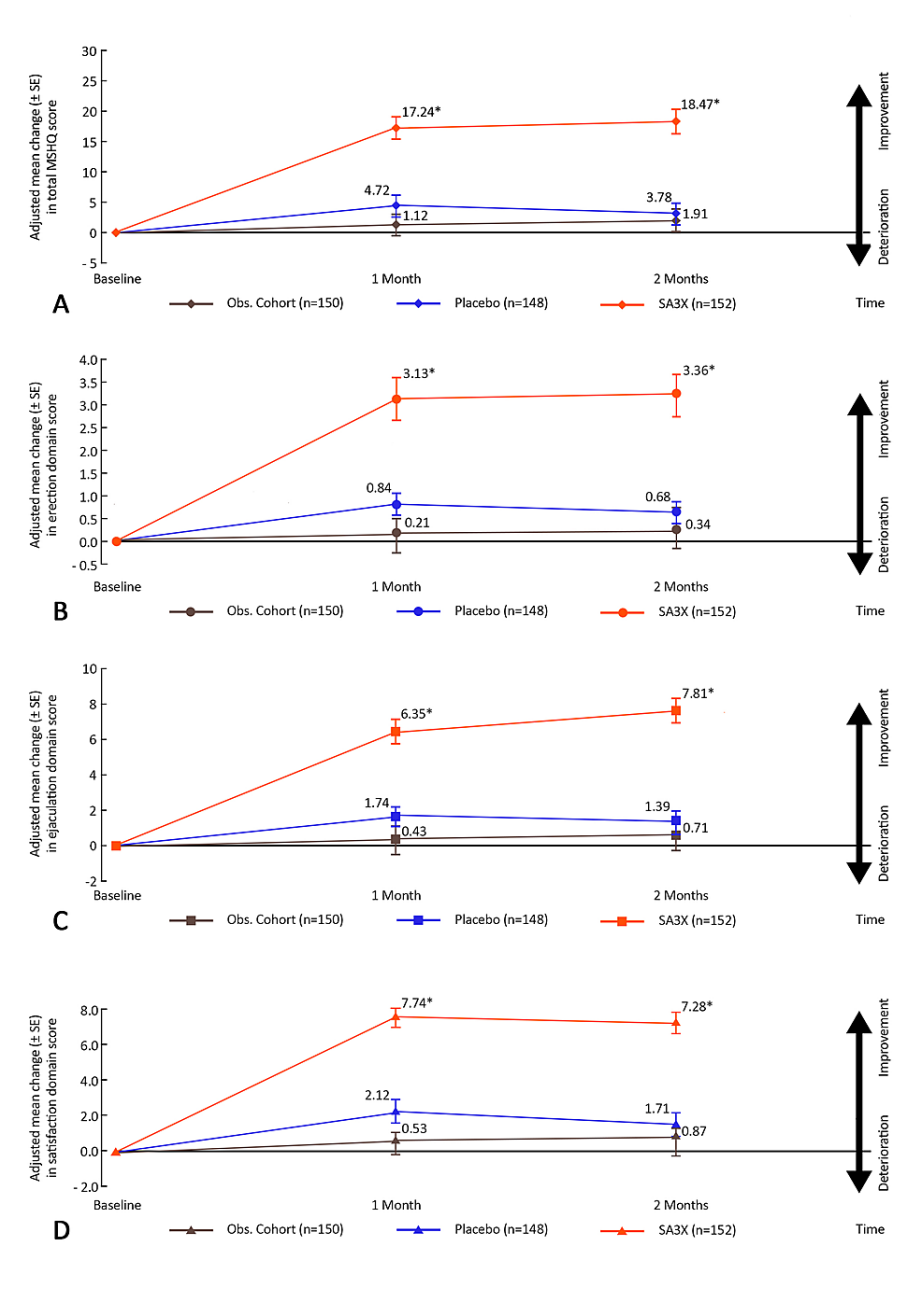
Adjusted mean (±SE) change in: (A) total MSHQ score, (B) erection domain score, (C) ejaculation domain score, and (D) satisfaction domain score from baseline to day-30 of treatment and one-month post-treatment (ITT population). ITT: Intention to Treat; MSHQ: Male Sexual Health Questionnaire; Obs. Cohort: Observational Cohort *P<0.05

SA3X therapy resulted in a statistically significant (P<0.001) improvement in total MSHQ score after 30 days of therapy with an adjusted mean change from baseline of 17.24 (standard error [SE] 2.11) in the SA3X therapy group vs 4.72 (1.98) in the placebo group and 1.12 (1.05) in the observational cohort (Figure 2A). Similar findings were noted among the three domains of MSHQ (Figures 2B-2D).

A greater change in mean MSHQ total score was seen from baseline to 30 days with SA3X therapy (baseline: 20.11, after therapy: 37.35) compared with placebo (baseline: 20.77, after therapy: 25.49) and observational cohort (baseline: 20.33, after therapy: 21.50). On MMRM analysis, the treatment difference between SA3X therapy group and placebo group in regards to the total MSHQ score after 30 days was found to be statistically significant (estimate - 12.51 ± 2.65; P<0.001). The same was observed across the three domains of MSHQ - erection domain score (estimate - 2.27 ± 0.91, P=0.02), ejaculation domain score (estimate - 4.62 ± 1.16; P=0.03) and satisfaction domain score (estimate - 5.62 ± 1.11; P=0.01). The other domains of MSHQ - bother, activity and desire - also showed a significant increase as highlighted in Table 3.

**Table 3:** Summary for MMRM analysis for change from baseline in total MSHQ scores and scores for the MSHQ domains (erection, ejaculation, satisfaction, activity, and desire score) (ITT population) MMRM: Mixed Model Repeated Measures; ITT: Intention to Treat; MSHQ: Male Sexual Health Questionnaire; IIEF: International Index of Erectile Function *P<0.05 † - P-value for the estimate of treatment difference between SA3X therapy and placebo ‡ - P-value for the estimate of treatment difference between SA3X therapy and no treatment

	Visit	Treatment Difference (SA3X therapy vs placebo)		Treatment Difference (SA3X therapy vs no treatment)	
		Estimate (Mean ± SD)	p-value†	Estimate (Mean ± SD)	p-value‡
Total MSHQ score	After 30 days of therapy	12.51 ± 2.65	<0.001*	14.62 ± 2.87	<0.001*
	At 1 month post-treatment	14.65 ± 2.49	<0.001*	16.58 ± 2.85	<0.001*
Erection Domain Score	After 30 days of therapy	2.27 ± 0.91	0.02*	2.91 ± 0.86	0.03*
	At 1 month post-treatment	2.65 ± 0.92	0.01*	3.01 ± 0.83	0.02*
Ejaculation Domain Score	After 30 days of therapy	4.62 ± 1.16	0.03*	5.93 ± 1.32	0.02*
	At 1 month post-treatment	6.41 ± 1.26	0.02*	7.12 ± 1.37	0.03*
Satisfaction Domain Score	After 30 days of therapy	5.62 ± 1.11	0.01*	7.21 ± 0.97	0.03*
	At 1 month post-treatment	5.56 ± 1.21	0.02*	6.42 ± 1.05	0.03*
Bother Domain score	After 30 days of therapy	4.01 ± 1.43	0.01*	4.35 ± 1.35	0.01*
	At 1 month post-treatment	4.21 ± 1.29	0.01*	4.42 ± 1.18	0.01*
Activity Domain score	After 30 days of therapy	5.56 ± 1.67	0.02*	6.32 ± 1.54	<0.001*
	At 1 month post-treatment	6.06 ± 1.43	0.03*	6.62 ± 1.57	<0.001*
Desire Domain score	After 30 days of therapy	6.18 ± 1.93	<0.001*	7.22 ± 1.77	0.03*
	At 1 month post-treatment	6.05 ± 1.21	0.01*	7.47 ± 0.93	0.02*
IIEF	After 30 days of therapy	15.48 ± 2.96	<0.001*	16.23 ± 2.68	<0.001*
	At 1 month post-treatment	16.43 ± 2.76	<0.001*	17.21 ± 3.01	<0.001*
Duration of penile erection (minutes)	After 30 days of therapy	7.39 ± 2.42	<0.001*	7.63 ± 2.58	<0.001*
	At 1 month f post-treatment	7.92 ± 2.21	<0.001*	8.02 ± 2.11	<0.001*

Secondary Efficacy Results

At month 1 post-treatment, SA3X therapy resulted in a statistically significant (P<0.001) improvement in the total MSHQ score with an adjusted mean change from baseline of 18.48 (2.81) in the SA3X therapy group vs 3.78 (1.39) in the placebo group and 1.92 (1.13) in the observational cohort (Figure 2A). Similar findings were also noted among the three domains of MSHQ (Figures 2B-2D). MMRM analysis also reflected a significant treatment difference SA3X therapy group and placebo group in regards to the total MSHQ score and its sub-domains as featured in Table 3.

**Figure 3:**
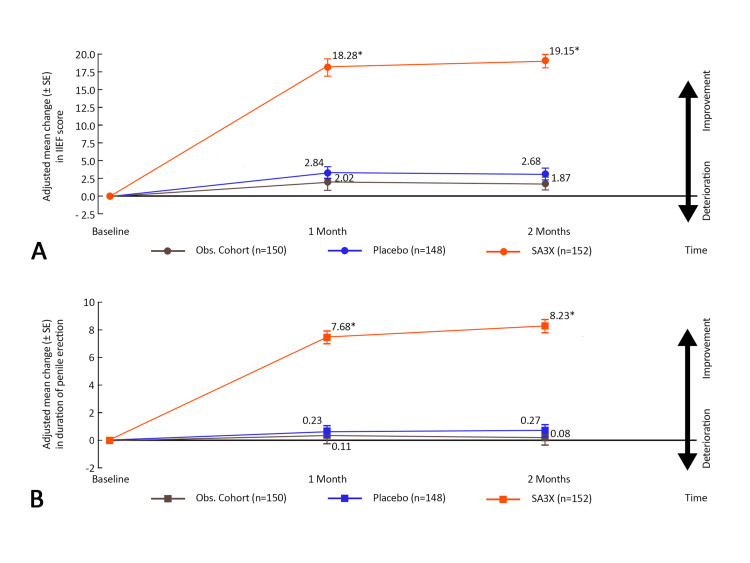
Adjusted mean (±SE) change in: (A) total IIEF score and (B) duration of penile erection, from baseline to day-30 of treatment and one-month post-treatment (ITT population). ITT: Intention to Treat; IIEF: International Index of Erectile Function; Obs. Cohort: Observational Cohort *P<0.05

Patients in the SA3X therapy group showed statistically significant greater improvements in IIEF score with an adjusted mean change from baseline of 18.28 (2.37) compared with placebo (2.84 [0.91]) and observational cohort (2.02 [0.76]) (Figure 3A).

Statistically significant greater improvements in IIEF score were seen in the SA3X therapy group compared with placebo after 30 days of treatment (P<0.001) as well as at one-month post-treatment (P<0.001), as was the case when compared with observational cohort (Table 3).

Patients in the SA3X therapy group had statistically significant improvement in the duration of penile erection as compared to baseline - adjusted mean change was 7.68 (0.64) minutes (Figure 3B) and the treatment difference when compared to placebo was also found to be statistically significant (P<0.001) (Table 3).

Safety

The proportion of patients with any AEs, SAEs, and drug related AEs was almost similar in the SA3X therapy group and in the placebo group. The most common drug-related AE was found to be dysgeusia (3.94%) among the SA3X therapy group (Table 4).

**Table 4:** Summary of adverse events in ITT population ITT: Intention to treat *P<0.05 † - test of significance used is Fischer Exact test

	Total (N = 298)	SA3X therapy (N = 152)	Placebo (N = 146)	P-value†
Fever	7 (2.34 %)	4 (2.63 %)	3 (2.05 %)	1
Nausea/ Vomiting	8 (2.68 %)	5 (3.28 %)	3 (2.05 %)	0.72
Dizziness	6 (2.01 %)	4 (2.63 %)	2 (1.37 %)	0.68
Dysgeusia	6 (2.01 %)	6 (3.94 %)	0 (0.00 %)	0.02*
Retrograde Ejaculation	1 (0.33 %)	0 (0.00 %)	1 (0.68 %)	0.48
Ejaculation Disorder	2 (0.67 %)	1 (0.66 %)	1 (0.68 %)	1
Ejaculation failure	1 (0.33 %)	0 (0.00 %)	1 (0.68 %)	0.48
Decreased semen volume	3 (1.01 %)	1 (0.66 %)	2 (1.37 %)	0.61

No SAEs were reported in this study. Only one patient developing dysgeusia discontinued medication and withdrew from the study. At the end of treatment and follow-up, only one patient in the SA3X group had unresolved ejaculation failure and another patient of placebo group complained of decreased seminal volume.

## Discussion

The current study is the first to use validated numerical scores to assess the domains of sexual function in men with ED treated with SA3X therapy. Our trial found an increase of 85.72% in MSHQ score from baseline among male participants receiving SA3X therapy for 30 days. The change in the total MSHQ score appeared to be driven largely by changes in the scores for the erection domain, which increased by almost 90% on average from baseline in the SA3X therapy group (P<0.001). Notably, after cessation of therapy, the effect seemed to be consistent with the MSHQ score showing a 91.89% increase at the end of one month, though the percentage increase is unlikely to be clinically relevant. However, the scores in the ejaculation domain increased by almost 100% at the end of the follow-up. In contrast, the absolute changes from baseline in the placebo and the no-treatment group were statistically non-significant.

Sharma et al. had proved the association of spilanthol with improved sexual performance in male rats, which reverberated with the findings of the current study [3]. Apart from the erection and ejaculation domains, a consistent increase was also noted in the satisfaction score in the SA3X therapy group along with improved scores in bother, activity, and desire domains. Dubey et al. reported the aphrodisiac properties [8] and increased transdermal absorption of testosterone was also noted by De Spiegeleer et al. due to spilanthol [17]. The present study also detected an increased duration of penile erection. However, given the various psychological and physical factors that influence sexual desire and satisfaction, it is obscure as to why such a phenomenon has been observed and needs further biochemical investigations.

Although plant-derived compounds such as yohimbine, *Panax ginseng*, *Butea superba*, Epimedium herbs (icariin), *Tribulus terrestris*, *Piper guineense* and *Lepidium meyenii* (Maca) have been extensively used for their prosexual effects, [18-26] they have been frequently studied on animals with human studies being scarce having insubstantial methodology and outcomes [1]. The same has been the case with Spilanthes with no registered clinical trials as yet. Surprisingly, despite limited scientific evidence, the demand for traditional medicines has been exorbitant which has been attributed to a positive attitude towards complementary medicines, lesser costs than medicine-based health services, cultural and historic influences, etc. [27,28]. Out-of-pocket expenditure for alternative medicines in the United States accounted to US$14.8 billion in 2008 and was expected to rise even more in recent years [29]. The same was the case with other Southeast Asian and African countries [27], which emphasizes the need to conduct clinical trials invariably geared towards safe and effective practices in traditional/ herbal supplementation.

The SA3X capsules were also found relatively safer with dysgeusia being the sole AE in the treatment arm which was significantly more in comparison to placebo group. Unlike other herbal compounds like yohimbine and *Tribulus terrestris*, SA3X did not lead to an increased blood pressure, stomach upset, tremors, or sleep issues [24,30]. Even so, analysis of biochemical and hormonal parameters will be required to ascertain long-term effects of SA3X capsules on humans.

Certain limitations which might affect the results of the current study are the patients being randomized from a single hospital, subjective assessment of some parameters and relying on self-reported data of patients. Regardless, the RCT is the first to evaluate the effect of *S. acmella *on humans with a stringent methodology with no cross-over of participants. The next step would be a follow-up study to investigate the hormonal parameters to verify the safety of the treatment. Though SA3X may not be considered a first-line treatment option for ED, its effectiveness and probability to be used in combination with other phosphodiesterase inhibitors cannot be overlooked.

## Conclusions

SA3X capsules have clinically proven to be an effective therapy to improve the sexual health of males in the present study. Furthermore, it is relatively safe with minimal side effects and also seems to improve the psychogenic domains of participants. Though the generalizability of the findings requires further scientific evidence in varied geographical strata and different races and cultures, nevertheless it can be considered a potential therapeutic intervention for ED.
